# Supplementary material for: A Cellular Assay for Spike/ACE2 Fusion: Quantification of Fusion-Inhibitory Antibodies after COVID-19 and Vaccination
Source: Viruses. 2022 Sep 25;14(10):2118. doi: 10.3390/v14102118 (PMC9609042; doi:10.3390/v14102118)
Supplement: Supplementary file 1 [file viruses-14-02118-s001.zip › Figure S1.pdf]

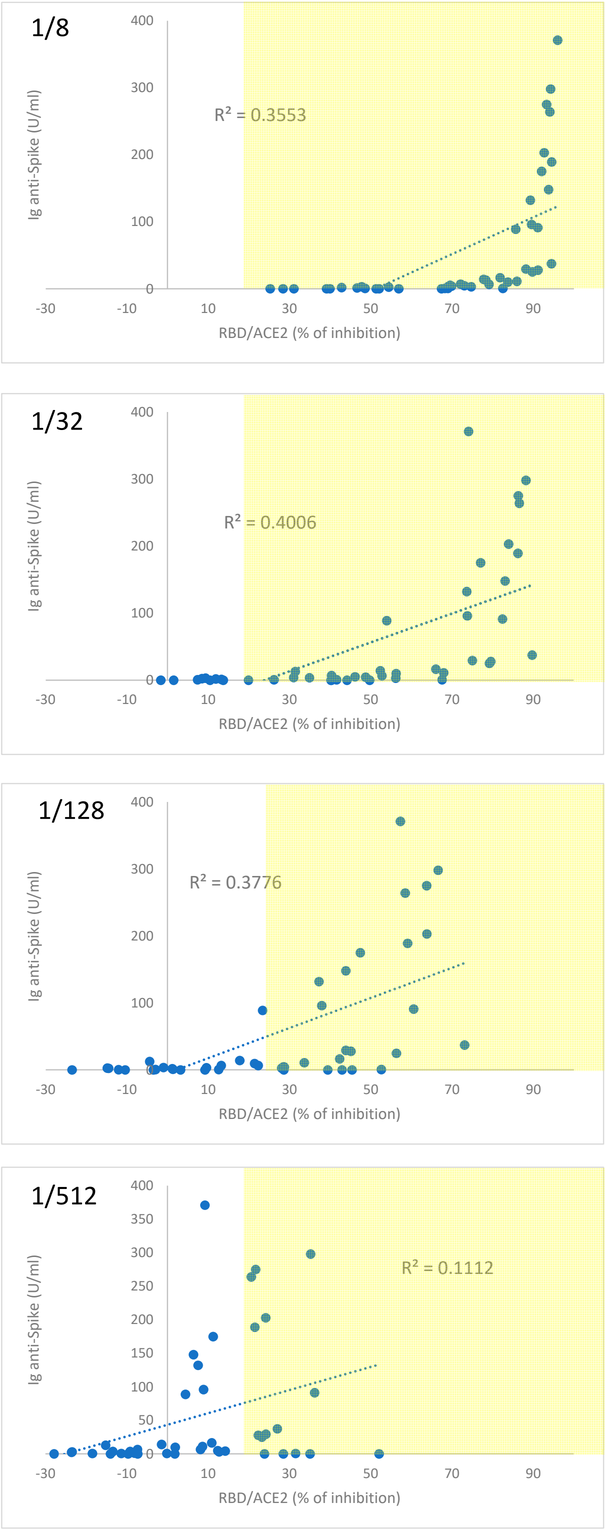

Figure S1. Comparison of the fusion-based assay with serology.

Forty-four sera were either analysed, for each dilution, with the fusion-based assay or the anti SARS-CoV-2 IgG quantitative ECLIA kit (Roche Diagnostics International Ltd, Rotkreuz, Switzerland)
