# Supplementary material for: A Cellular Assay for Spike/ACE2 Fusion: Quantification of Fusion-Inhibitory Antibodies after COVID-19 and Vaccination
Source: Viruses. 2022 Sep 25;14(10):2118. doi: 10.3390/v14102118 (PMC9609042; doi:10.3390/v14102118)
Supplement: Supplementary file 1 [file viruses-14-02118-s001.zip › Table S1.pdf]

t (William’s test)	fusion/ELISA<br>serology/ELISA	serology/ELISA<br>fusion/serology	fusion/serology<br>fusion/ELISA
1/8	0.35	0.38	0.95
1/32	0.37	0.48	0.85
1/128	0.53	0.29	0.67

Table S1. Statistical comparison (William’s t test) of the correlation coefficients calculated from the different comparisons among the three methods. The t value was calculated by the cocor test (http://comparingcorrelations.org). A significant difference between correlation coefficients is demonstrated by a $t<0.05$.
